# The Geography of Fast Food Outlets: A Review

**DOI:** 10.3390/ijerph7052290

**Published:** 2010-05-06

**Authors:** Lorna K. Fraser, Kimberly L. Edwards, Janet Cade, Graham P. Clarke

**Affiliations:** 1 School of Geography, University of Leeds, LS2 9JT, UK; E-Mail: g.p.clarke@leeds.ac.uk; 2 Cancer Epidemiology Group, Division of Epidemiology, University of Leeds, LS2 9JT, UK; E-Mail: k.l.edwards@leeds.ac.uk; 3 Nutritional Epidemiology Group, Division of Epidemiology, University of Leeds, LS2 9JT, UK; E-Mail: j.e.cade@leeds.ac.uk

**Keywords:** fast food, obesity, take away, geography

## Abstract

The availability of food high in fat, salt and sugar through Fast Food (FF) or takeaway outlets, is implicated in the causal pathway for the obesity epidemic. This review aims to summarise this body of research and highlight areas for future work. Thirty three studies were found that had assessed the geography of these outlets. Fourteen studies showed a positive association between availability of FF outlets and increasing deprivation. Another 13 studies also included overweight or obesity data and showed conflicting results between obesity/overweight and FF outlet availability. There is some evidence that FF availability is associated with lower fruit and vegetable intake. There is potential for land use policies to have an influence on the location of new FF outlets. Further research should incorporate good quality data on FF consumption, weight and physical activity.

## 1. Introduction

One of the factors implicated in the obesity epidemic is the availability of inexpensive and unhealthy food. Fast Food (FF) has its roots in Southern California in the 1940s but the number of Fast Food outlets has increased dramatically and McDonalds alone owns >30,000 outlets worldwide [1] and the average US citizen eats 3 burgers and 4 portions of French fries per week [2]. The Fast Food industry has been so successful due to the fact that it is quick, convenient and uniform in its production (DeMaria 2003). Children ate 300% more food from restaurants and Fast Food outlets in 1996 than in 1977. This may be due to several changes such as two parents working so less time to cook for family, relatively cheap Fast Food and food advertising [3].

The Fast Food and takeaway market in the UK was worth £8.9 billion in 2005 with predictions of steady 5% annual increases likely [4].The birth of the Fast Food (Fast Food) restaurant and the exponential growth of that industry almost parallels the obesity epidemic, certainly in the western world [5,6].

Fast Food is known to be energy dense, high in saturated fat and have low micronutrient content [7–12] and its consumption is associated with other poor food choices such as low vegetable and milk intake [13]. The consumption of fast food has been associated with increased body mass index (BMI) and obesity [14,15]. This consumption is in part due to an individual or family’s eating behaviour but in the last decade there has been a realisation that micro and macro environmental factors may as also be important in the obesity epidemic [16]. The ‘obesogenic environment’ [16] is used to describe modern societies where the availability of green spaces and leisure facilities is poor and the availability of unhealthy foods is good. The ‘food environment’ body of research has incorporated studies of potential ‘food deserts’ as well as availability and access to healthy and unhealthy foods. The location of FF outlets and therefore the availability of such foods to the population has been a recent research interest and this review aims to summarise the research to date and the implications of this and to identify potential areas for future research.

## 2. Methods

A semi-systematic review was undertaken. Medline, Embase and Web of Science were searched for the period from 1990 until April 2009. The abstracts of all identified papers were then examined and only studies which fitted the inclusion criteria (see below) were included. The full text article was obtained for those studies which fitted the criteria. Hand searching of all references from included papers was also undertaken.

Search Strategy: Search terms used were ‘Fast Food’, ‘takeaway’, ‘take-away’, and ‘food outlets’. There was no restriction on study type or language.

Study Selection: The inclusion criteria specified that the published material reviewed needed to have studied the geographical location of fast-food or takeaway outlets.

Data Extraction: A data extraction form was completed for each of the included studies which collected data on:

1. Study Design
2. Fast Food outlet definition
3. Other Food outlets included
4. Geographical setting
5. Food outlet data sources
6. Availability of overweight/obesity status
7. Food consumption data
8. Outcomes: BMI, fruit and vegetable intake.
9. Analyses

Synthesis: The results were analysed by study design type however a meta-analysis could not be performed due to the heterogeneity of the studies.

## 3. Results

Initial searching resulted in the assessment of 447 abstracts. Full text of 48 papers which potentially would fit the inclusion criteria were obtained and upon closer examination, 33 of these papers met the inclusion criteria, so were included in the final analysis. No additional papers were determined from the references contained within these 33 papers.

16 of the 33 studies used a population level approach (*i.e*., data pertained to entire cities or communities within cities) and the other 17 used individual level data (see Table 1). Three of the population level studies concentrated on fast food availability around schools whilst four of the individual level papers included data on children and fast food access.

The studies were heterogeneous in their definitions and analysis as discussed below (summarised in Table 1).

### 3.1. Fast Food Outlet Definition

The majority (n = 26) of the studies used a narrow definition of FF outlets, which included only major national or international franchises. One of these, an early study by Cummins *et al*. [18], included only McDonald’s outlets. Just five of the studies [19–23], undertaken more recently, used a broader definition including small independent outlets as well as the major franchises. Two of the studies had no definition of FF stated in their papers [24,25].

### 3.2. Availability of other Food Outlets

Twenty one of the studies used data and analysis based on other food outlets: supermarkets [19,23,27]; convenience stores [23,27,28]; full service restaurants [7,24,27,29–34]; or all food outlets [25,35–39].

### 3.3. Geographical Setting

The majority of studies to date have been undertaken in the United States (n = 21). Of these, six concentrated on large cities only [7,22,30,37,40,41], one looked at only rural areas [25], and the rest had an urban/rural mix [28–29, 31–33,35–36,38–39, 42–46,]. There have been six studies from Australia (one rural [24]/five in cities [20,26–27,47,48] and two national studies from New Zealand [21,23]. The three studies from the United Kingdom were all performed by the same research group; one was a study restricted to Glasgow [32]. Whilst the other two looked nationally at England and Scotland at the population level [18,49]. The other study was from Canada [19]. The geographic areas in which data was collected and analysed varied between studies from super output areas [49] to state level [42].

### 3.4. Food Outlet Data Sources

The majority (n = 23) of these studies identified FF outlets (and any other food outlets included) via a single data source. These included local authority or government databases (n = 15) as well as industry owned databases (n = 8). Seven of the studies used telephone directories. Only six of the studies [21,23,27,39,40,44] stated that they used a secondary source to cross check findings such as the online yellow pages. Data validation, such as physically visiting the study area by car to confirm the existence of such stores, was only performed in one study [20] where they visited the whole study area. There is, however, no discussion of the accuracy of their electronic data after this ground-truthing had been undertaken.

### 3.5. Assessment of Overweight/Obesity

Fourteen of the studies included a measure of weight and height and therefore overweight or obesity status (two of which focused solely on children): one was population level and used state level obesity rates [42]; eight used self-reported heights and weights [25,29–31,33,36,39,43]; another five used measured weights [21,24,37,38,46].

### 3.6. Food Consumption Data

Only nine studies had food consumption data to incorporate into their analyses. Three of these used fruit and vegetable consumption as inverse proxies for FF consumption [21,27,29]. The other six used a measure of frequency of FF consumption [20,24,31,43,45,48], in each case asking a sample of the population about the frequency of eating at FF outlets or takeaways in the last month.

### 3.7. Analyses

#### 3.7.1. Accessibility measures

The studies which looked at proximity of FF outlets to home and/or work (n = 12) used mean or median distances. Most of these distances were Euclidian (straight line) distances, which take no account of road networks. One study used the cost surface (actual distance travelled) methods for walking, driving and public transport [26] and another three studies used network street distances [19,36,46]. Another proximity measure used by six studies was to draw ’buffers’ around centroids of small geographical areas or around the schools. The buffer distances used were variable which makes comparison between studies difficult. 400 m and 800 m were most often used as were 500 m/1,000 m/1,500 m and measures in miles (0.5, 1). Very few studies looked at distances greater than 1,500m. Density measures were also used: *i.e*., number of FF outlets per geographic area were, used by 22 of the studies (see Table 1).

#### 3.7.2. Statistical approaches

The majority of studies used simple statistical techniques such as correlation or simple regression modelling to look at the association between density and/or proximity of FF outlets, socioeconomic factors and/or weight status. Only five studies used multilevel modelling to take into account individual and area level factors [21,22,30,33,39]. One study [40] used the K clustering analysis to assess whether there was clustering of FF outlets around schools.

### 3.8. Study Results

The study results are summarised by outcome and study type in Table 2. The majority (n = 14) of the 16 studies which looked at an entire population showed a significant association between increasing area level deprivation levels variables and the availability fast food outlets. *i.e*., income; decreased income, increased FF exposure [7,19,28,41,44,47,48], socio-economic status; increased deprivation, increased FF exposure [18,23,26,48,49], ethnicity [7,22] and FF exposure (measured by proximity to home/school/work or density by area). Only two studies showed no association [32] between socioeconomic status proxies and FF exposure. .

The studies within Scotland and England showed that in England there is a positive linear relationship between the density of McDonald’s outlets and deprivation. In Scotland this trend was similar, except that the highest FF outlet density was found in the second most deprived quintile, not the most deprived quintile [18]. These results were replicated when the study was repeated with the addition of three other major franchise chains (Pizza Hut, Burger King, and Kentucky Fried Chicken) [49]. Interestingly when this group focused solely on Glasgow and included all food outlets, they found no association with socioeconomic status (measured using the Scottish Index of Multiple Deprivation). In fact, 50% of the FF outlets in Glasgow were in the second most affluent quintile [32].

The one ecological study [42] with BMI data found a positive association between the density of FF outlets per square mile and obesity rates.

To date these ecological results have, largely, not been verified with the results from studies using individual level data. Of the six individual studies which had a measure of deprivation only two [46,48] found a positive association between increasing deprivation levels and FF exposure. The other four studies found no association [20,30,39,41].

The evidence for an association with FF outlet availability and obesity is weaker, of the 12 cross-sectional studies which looked at FF outlets in relation to overweight or obesity, six found a significant positive association [29–31,33,36,43], two had significant negative results [21,36] and five showed no association [24,25,37,39,46]. Of the studies which showed a positive association between FF outlets and weight/BMI, one only found an association in non-car owners [30], one found an association in adult females only [43], one found a significant association between increased number of FF outlets and increased obesity but also decreased obesity if closer to a FF outlet [36], and one found an association between weight status and FF exposure in schools [29]. The other study with a positive result [33] aggregated their individual level data to perform a county level analysis. All six of these studies used self-reported heights and weights to calculate BMI. The longitudinal study found no association between density of FF outlets and BMI change in children [38].

The six studies which have incorporated FF consumption data have conflicting results; three had some positive associations between FF outlet availability and FF consumption [43,45,48], and three had no association [20,24,31]. Jeffery [31] found a positive association between frequency of FF consumption and BMI but no association between FF consumption and FF exposure. One of the positive studies which used FF consumption frequency found that increased exposure to FF outlets increased consumption by 11–61% in adults [45]. The three studies which used fruit and vegetable consumption as an inverse proxy for FF consumption all found that having increased FF availability decreased your fruit [21,27,29] and vegetable [29] intake.

Three of the four studies which looked at the location of Fast Food outlets in relation to schools were ecological in design. Two of these found a significant positive association between deprivation and FF availability [28,41], the other study found no association with deprivation but did find clustering of FF outlets around schools [40]. The other study was cross sectional and it found that children who attended schools with greater availability of FF outlets had increased weight compared with schools with fewer FF outlets [29].

## 4. Discussion

There are a large number of studies which have shown a significant relationship between lower area level socioeconomic status and higher availability of FF outlets. The cross sectional studies have shown mixed results for the association between FF availability and weight status but there is some evidence that greater exposure to FF is associated with a lower fruit and vegetable intake.

The conflicting results from the studies could be partly explained by a number of methodological issues:

### 4.1. Fast Food Definition

The implications of including only major franchises are obvious; the total number of outlets will decrease and therefore false positive or false negative associations may be found. To try to assess true associations between the location of FF outlets and weight status, all outlets which sell typical FF (burgers, pizza, chips *etc*.) need to be included in the analysis.

### 4.2. Availability of Other Outlets

There is an issue with the studies which looked at FF outlets alone. Not including all food outlets (supermarkets, restaurants, convenience stores *etc*.) means that these studies cannot account for the availability of choice. People may eat at a FF outlet simply because there is no alternative food outlet nearby. This is an easier area to address with policy decisions than if people are chosing to eat at FF outlets rather than healthier alternatives. All the studies used other food outlets as alternatives to FF outlets as opposed to other places where FF could be consumed. Ideally the foods available from the included outlets would be checked but this can be very time consuming.

### 4.3. Food Outlet Data Sources

As there was little information given on the known accuracy of the electronic databases, this was a source of potential measurement error. These databases are only accurate on the day the data are collected and may go out of date quickly. The lack of physical validation of the existence of the food outlets is a limitation of nearly all the studies. This suggest that ground-truthing of at least a sample of the study area is crucial to validate the food outlet data.

### 4.4. Setting

Although most of the studies were undertaken in the USA, there are studies from the UK and the southern hemisphere, so generalising t he results to most western countries may be valid. However, studies from other European countries with different eating cultures would be welcome. Research into the availability of FF and its relationship with weight in the developing world where the dual burden of malnutrition and obesity is evident would be valuable.

### 4.5. Assessment of Overweight/Obesity

Interestingly all the studies that found a significant positive association between FF availability/exposure and overweight or obesity used self-reported weights. Self reported weights are known to be prone to bias [50] but this would usually be underreporting of weight which would be unlikely to account for these findings. Ideally heights and weights should be measured by trained individuals using validated equipment but this may not be feasible due to available resources All apart from one of the studies were cross-sectional in nature and therefore cannot confirm causality. Further longitudinal studies may help clarify the relationship between the availability of Fast Food and overweight/obesity.

### 4.6. Fast Food Consumption

Whilst FF consumption has been shown to be associated with decreased fruit and vegetable intake, using fruit and vegetable intake as an inverse proxy for FF consumption is not ideal [51]. The studies which asked a question about frequency of consumption at FF restaurants have used a more valid measure of FF consumption than the fruit and vegetable example but there was no attempt in any of the studies to ascertain what foods were eaten at the FF outlet. This is important as some ‘healthier’ alternatives are now available in FF outlets [52]. Knowing the actual foods consumed would allow analyses on different types of FF; burgers, pizza, curry *etc*. The use of food diaries or a full FFQ would inform on both the amount of FF eaten and the effect on the overall diet.

### 4.7. Spatial Scale

The geographical scale used for analyses in these studies varied from small areas (*i.e*., census blocks) to larger areas (*i.e*., county level). Using small area geographical analysis allows areas with higher ‘risk factors’ or ‘disease prevalence’ to be identified. Using larger areas for analysis results in these small areas of high or low ‘risk’ being averaged out and thus a loss of information [53].

### 4.8. Analyses

#### 4.8.1. Measuring access

Most of the studies used straight line distance which is an unrealistic measure of access. Using network distance for analyses is a more realistic measure as most people cannot travel to their nearest FF outlet in a straight line but there are more sophisticated measures of ‘access’ such as spatial interaction modelling [54]. As well as distance between home and destination, this type of modelling accounts for the ‘attractiveness’ of the food outlet.

Only five of the studies used multilevel modelling techniques in their analyses. In this type of analyses individual level variable and area level variables are not independent of each other and therefore traditional regression modelling techniques should not be used. Other statistical approaches such as geographically weighted regression may also be useful in this field of research.

The use of spatial microsimulation (SM) modelling should be explored in this field of work [55]. SM involves building spatially disaggregated large-scale micro-datasets on the attributes of individuals or households, often using a combination of information sources (such as, census data, hospital records, surveys). Its main benefit is that it can estimate the geographical distribution of variables which were previously unknown: for example the distribution of obese children across households in a city [54].

#### 4.8.2. Implications of the study results

The results from the ecological studies show that there are more FF and takeaway outlets in more deprived areas. This may be an example of the ‘deprivation amplification’ effect where residents in deprived areas have poorer access to health promoting resources than more residents in more affluent areas [56] but most of these studies have not commented upon the availability of healthy food outlets. These results have started to change policy; in 2008 the City of Los Angeles passed a bill to ban the opening of any Fast Food restaurants in the poor neighbourhoods in the city. These studies are from USA, UK, Australia and Canada so these results may be generalisable to the Western world.

All the studies which showed a positive association between FF availability and overweight/obesity were undertaken in the USA, in fact only 2 of the studies which had weight status as an outcome were undertaken outside of the USA. There is a need for studies from other countries with good quality height and weight data to be undertaken.

Six out of the nine studies which looked at food consumption in relation to the availability/location of FF outlets found a significant association in the expected direction. The finding that the increased availability of FF outlets is associated with poor food choices (decreased fruit intake and higher fast food intake) is interesting but in order to fully assess the potential health consequences more dietary data is required. Obtaining accurate dietary data is difficult and time consuming but the use of food diaries or full FFQ to describe the whole diet of participants could allow for an increased understanding of the potential implications of increased availability of FF outlets.

The studies which looked at schools found that schools have more FF outlets in close vicinity than would be expected by chance and the majority found that this was amplified in more deprived areas. Whilst only four studies were undertaken in schools this is important information for planning authorities to take into consideration; in London, the Waltham forest council have banned any new Fast Food outlets opening within 400m of their schools. This policy measure could be used more widely to help reduce children’s exposure to FF.

### 4.9. Confounding Factors

Physical Activity is a very important potential confounder in the studies which used weight status as an outcome. Only one study adjusted for physical activity levels in their analyses [31]. This study also adjusted for the number of hours watching television which has been shown to be an independent risk factor for obesity [57]. Another potential confounder is car access. Although many of the studies have used distances able to be walked in 5 to 10 minutes this does not account for the people who drive 5 or 10 minutes to a FF outlet from home or work. None of the studies adjusted for car access or home delivery of FF.

There are many other potential confounding factors in the association between FF outlets and obesity that were not considered in any of the studies, such as parental eating habits, parental physical activity levels and parental obesity.

## 5. Conclusions

There is a growing body of literature assessing the geography of FF outlets, especially in association with overweight and obesity Most of the studies have found a positive association between availability (proximity and density) of FF outlets and increasing deprivation. This may be due to the companies targeting more deprived areas as the land is cheaper or it may be that the demand from consumers in these areas is greater. Either way this is an important issue to highlight to policy decision makers as land use restrictions on new Fast Food outlets may help to stop the ‘deprivation amplification’ effect.

The association between availability of FF outlets and overweight/obesity status is less clear as there have been conflicting results. The studies looking at association between the consumption of FF and the exposure to FF outlets have also found conflicting results which may be due to the lack of good quality dietary information. The results show that children in schools are exposed to more FF outlets than expected and this has important policy implications.

There is a need for research which combines good methodology with data on as many possible potential confounding factors. The geographical analysis should combine the exposure to FF outlets with consumption data as well as physical activity and transport data.

## Figures and Tables

**Table 1.** Summary of Included Studies and Methodologies.

*Author/year/location/design (E = Ecological, X = Cross sectional, L = Longitudinal)*	*Participants*	*Fast-Food definition*	*Outlet Identification*	*Other Food Outlets*	*Weight Status*	*Food Consumption*	*Geographic Scale*	*Analysis*
Austin 2005 USA(Chicago) E	1,292 schools	Eating places where customers order items & pay before eating and has eat out option.	Commercial database. Validated with yellow pages.	None	None	None	Census Tracts	Number FF < 400m < 800 m schools (buffers). Mean/median distance to FF.
Blair-Lewis 2005 USA (Los Angeles) E		NAICS*	Environmental Health Database	Restaurants	None	None	Zip Code level.	Zip code density full/limited service rest
Block 2004 USA (New Orleans) E		Chain restaurants > 2 of; expedited food, takeout, limited/no wait staff, pay first.	Council Log Book, Yellow Pages	None	None	None	Census Tracts and “shopping area”	Census tract, “shopping area” 1 mile buffer. Number FF per square mile.
Burdette 2004 USA (Cincinatti) X	7,020, 3 and 4 year olds from low income households	All franchises (national)	Yellow Pages	None	Measure d	None	‘neighbourhood’ not defined.	Mean street distance to FF outlet from home.
Burns 2007 Australia (Melbourne) E	180,000 population	Large franchises (> 10 outlets)	Council Database	Supermarkets.	None	None	Census Collected Districts	Cost surface measure of travel time by car, bus & walking to nearest FF and supermarket.
Casey 2009 USA (rural) E	1258 adults	Not Stated	N/A	Other Food stores	Self reported	None	N/A	Perceived access.
Cummins 2005 England/Scotland E		McDonald’s	Yellow Pages	None	None	None	SOAs and Data Zones	Mean number of FF per 1000 people per area.
Davis 2009 USA (California) X	500,000 youths School based	Top limited service restaurants	Commercial Database	Restaurants.	Self-reported	FFQ fruit veg soda.	0.5mile buffer of school.	FF rest within 0.5 mile of the school.
Frank 2009 USA (Atlanta) X	4,545 adults 25–60 years	Franchises	Manual review of names of outlets	Grocery stores.	Self Reported	Visits to FF outlets.	1 km road network distance buffer around home & work	Linear regression
Inagami 2009 USA (Los Angeles) X	2,156 adults	NAICS	Environmental Health Database.	Restaurants	Self reported	None	Census tract.	Density per roadway mile/census tract. MLM^#^
Jeffery 2006 USA (Minnesota) X	1,033 adults	SIC **	Commercial database.	Restaurants.	Self reported	Frequency of eating at FF outlets.	2 mile buffer of home.	Density 0.5 mile/1 mile/2 miles of work and home.
Kwate 2009 USA (New York) E		National & local chains that: No table service. Cash register / Drive through. Pay before eat. Burger, chicken, hot dogs.	Environmental Health Database.	None	None	None	Census Block	Grid 60 m^2^ number of FF < 300 m from centroid. Average exposure per block group.
Macdonald 2007 Scotland/England E		McDonald’s, Burger King, Pizza Hut, KFC	Yellow Pages and Burger King website.	None	None	None	SOAs and Data Zones.	Density per 1,000 population per SOA/DZ.
Macintyre 2005 Scotland (Glasgow) E		Burger King, McDonald’s, Pizza Hut, KFC, Wimpy	Council Database	Restaurant, Cafe	None	None	Data Zones	Mean number outlets per 1,000 population per data zone.
Maddock 2004 USA E		SIC	Yellow Pages	None	State level	None	State level	Density of FF outlets per square mile.
Mehta 2008 USA X	714,054 adults	Chains	Not Stated	Restaurants	Self-reported	None.	County Level	Number per 10000ind. Ratio FF/full service. MLM.
Moore 2009 USA X	5,633 adults	33 national franchises	Commercial databases	None	None	FFQ-fast food frequency	1 mile buffer.	Fast Food exposure = Self-report, informant report, GIS densities. 1 mile kernel densities
Morland 2002 USA (Mississippi) E		NAICS	Environmental Health database	All Food outlets	None	None	Census Tract	Number of food stores per census tracts.
Morland 2009 USA X	1,295 adults	NAICS	Environmental Health Database	All food outlets	Self reported	None	Census Tract.	Network distance & density per census tract.
Pearce 2007 New Zealand E		Multinational & local	Territorial Authority database. Validated with yellow pages.	Supermarkets, convenience stores.	None	None	Meshblock	Distance from centroid meshblock to FF outlet. Travel time. Schools access; dist
Pearce 2009 New Zealand X	12,529 people aged > 15 years	Multinational & local	Territorial Authority Database. Validated with yellow pages.	None	Measure d	FFQ fruit & Vegetables	Meshblock.	Multilevel model. Above/below averaged median distance per neighbourhood.
Powell 2007 USA E	99.8% population	SIC	Commercial Database.	Restaurants	None	None	Zip Codes	Zip codes densities.
Reidpath 2002 Australia (Melbourne) E		One of the largest FF chains e.g. MacDonald’s, Pizza hut, KFC	Yellow pages	None	None	None	Postal Districts	Density per postal district per population.
Rundle 2009 USA (New York) X	13102 adults	SIC	Commercial Database.	All Food outlets.	Measure d	None	0.5mile network buffer around home.	Density per square km by 0.5 buffer around home. MLM.
Simmons 2005 Australia X	1454 adults	Not Stated.	Telephone Directory	Restaurants	Measure d	Freq takeaway.	Per 1000 population.	Number of takeaways per 1000 population for town and restaurants.
Simon 2008 USA (Los Angeles) E	1684 schools	18 Fast Food chains	Council Database.	None	None	None	Census tract.	FF < 400 m < 800 m school (buffers)
Smoyer-Tomic 2008 Canada (Edmonton) E		Walk-up counter service selling predominantly pre-processed and prepared to order foods.	Council Health Inspection Database.	Supermarkets	None	None	Census Block	Network street dist 500/800/1,000/1,500m from geometric centre of census block. Density of FF < 500 m. Nearest distance also calculated.
Sturm 2005 USA L	13,282 children (4–7 years)	NAICS	US Census Business Directory	All food outlets	Measure d	None.	Zip code level	Density of FF outlets per zip code. MLM.
Thornton 2009 Australia (Melbourne) X	2,547 adults	Red Rooster, KFC McDonalds, Hungry Jacks, Pizza Hut	White Pages	None	None	How often purchase FF from any of the 5 franchises?	Census Collectors Districts.	Density; total number FF within 3 km road network from home Varity; same but number different FF outlets. Proximity; road network dist to nearest FF. MLM.
Timperio 2008 Australia X	1,001 children (aged 5–6 and 10–12 years)	8 commonest chains.	Council databases. Validated with yellow pages.	Convenience, greengrocer, supermarket, cafes, restaurants & takeaway.	None	FFQ fruit & veg	800 m network buffer of home.	Availability food outlets < 800 m home. Shortest road distance.
Turrell 2008 Australia (Brisbane) X	1,003 households	All Fast Food and takeaways.	Council database. Validated by groundtruthing	Cafe	None	Frequency takeaway	Census Collected District	2.5 km buffer density per centroid CCD. MLM
Wang 2007 USA X	7,595 adults	NAICS	Californian Stat Board and business telephone directories.	All food outlets	Self reported	None	Census Tract/Block	Density no. Per census tract + 0.5 mile buffer. Proximity, straight line distance. MLM
Zenk 2008 USA E	31,433 secondary schools	SIC	Commercial database.	Convenience Stores.	None	None	Census Tract	Number FF & con < 0.5 miles school. Number per census tract.

* NAICS North American Industry Classification System

** SIC Standard Industry Classification

# Multi level modelling.

**Table 2.** Summary of Study Results by Study Design and Outcomes.

	Significant Positive Association	Significant Negative Association	No Significant Association
**ECOLOGICAL STUDIES** (n = 16)			
Socioeconomic Status (n = 14)	Block 2004 Burns 2007 Morland 2002 Cummins 2005 MacDonald 2007 Pearce 2007 Powell 2007 Blair Lewis 2005 Reidpath 2002 Smoyer Tomic 2009 Simon 2008 Zenk 2008		Austin 2005 MacIntyre 2005
Ethnicity/Race (n = 2)	Kwate 2009 Smoyer Tomic 2009		
Weight (n = 1)	Maddock 2005		
**CROSS SECTIONAL STUDIES** (n = 16)			
Weight (n = 12)			
Self Reported (n = 8)	Mehta 2008 Frank 2009 (female only) Morland 2009 (density only) Jeffery 2006 Inagami 2009 (non car owner only) Davis 2009	Morland 2009 (proximity only)	Wang 2007 Casey 2009
Measured(n = 4)		Pearce 2009	Burdette 2004 Rundle 2009 Simmons 2005
Consumption (n = 9)			
Fast Food (n = 7)	Moore 2009 Frank 2009 (females only) Thornton 2009 (variety only)		Simmons 2005 Turrell 2008 Jeffery 2006
Fruit & Vegetables (n = 2)		Pearce 2009 (fruit only) Timperio 2009 (fruit only) Davis 2009 (fruit and vegetables)	
**LONGITUDINAL STUDIES** (n = 1)			
Weight			Sturm 2005
**SCHOOLS** (n = 4)			
ECOLOGICAL STUDIES (n = 3)			
Socioeconomic Status	Simon 2008 Zenk 2008		Austin 2005
Clustering	Austin2005		
CROSS SECTIONAL STUDIES (n = 1)			
Weight	Davis 2009		
